# The Cholesterol-Binding Sequence in Monomeric C-Reactive Protein Binds to the SARS-CoV-2 Spike Receptor-Binding Domain and Blocks Interaction With Angiotensin-Converting Enzyme 2

**DOI:** 10.3389/fimmu.2022.918731

**Published:** 2022-07-08

**Authors:** Hai-yun Li, Ning Gao, Cheng-yang Liu, Xiao-ling Liu, Feng Wu, Nini Dai, Jing Han, Qiu-yu Li

**Affiliations:** ^1^ Ministry of Education (MOE) Key Laboratory of Environment and Genes Related to Diseases, School of Basic Medical Sciences, Xi’an Jiaotong University, Xi’an, China; ^2^ Department of Biochemistry and Molecular Biology, School of Basic Medical Sciences, Xi’an Jiaotong University, Xi’an, China; ^3^ Department of Infectious Disease, the Second Affiliated Hospital of Xi’an Jiaotong University, Xi’an, China; ^4^ Academy for Advanced Interdisciplinary Studies, Peking University, Beijing, China; ^5^ Ministry of Education (MOE) Key Laboratory of Cell Activities and Stress Adaptations, School of Life Sciences, Lanzhou University, Lanzhou, China; ^6^ Center of Teaching and Experiment for Medical Post Graduates, School of Medicine, Xian Jiotong University, Xian, China; ^7^ Department of Respiratory and Critical Care Medicine, Peking University Third Hospital, Beijing, China

**Keywords:** SARS-CoV-2, monomeric C-reactive protein, pattern recognition receptor, ACE2, cholesterol-binding sequence

## Abstract

The receptor-binding domain (RBD) of the spike protein of severe acute respiratory syndrome coronavirus 2 (SARS-CoV-2) binds to the human angiotensin-converting enzyme 2 (ACE2) receptor, which is a prerequisite for the virus to enter the cell. C-reactive protein (CRP) is an important marker of inflammation and is a putative soluble pattern recognition receptor. Clinical elevation of CRP levels in patients with COVID-19 is one of the characteristics of the disease; however, whether CRP is involved in COVID-19 pathogenesis is unknown. Here, we report that monomeric CRP (mCRP) can bind to the SARS-CoV-2 spike RBD and competitively inhibit its binding to ACE2. Furthermore, truncated mutant peptide competition assays and surface plasmon resonance binding experiments showed that the cholesterol-binding sequence (CBS, amino acids 35-47) in mCRP was critical for mediating the binding of mCRP to spike RBD. In a cell model of spike RBD and ACE2 interaction, the CBS motif effectively reduced the binding of spike RBD to ACE2 overexpressed on the cell surface. Thus, this study highlights the pattern recognition function of mCRP in innate immunity and provides a preliminary theoretical basis for the development of the CBS motif in mCRP into a functional peptide with both diagnostic significance and potential therapeutic capabilities.

## Introduction

Coronavirus disease 2019 (COVID-19), caused by severe acute respiratory syndrome coronavirus 2 (SARS-CoV-2), is a serious threat to human health and public health safety, with pneumonia as the main clinical symptom (1, 2). The World Health Organization (WHO) declared COVID-19 a pandemic in March 2020. It has spread rapidly worldwide owing to its highly infectious nature and continues to pose a threat to public health and safety while causing enormous economic losses. Similar to other coronavirus infections, SARS-CoV-2 infection is initiated by binding of the spike (S) protein, a transmembrane glycoprotein that is exposed on the surface of viral particles and consists of two subunits, S1 and S2, to receptors on the surface of susceptible host cells (3). S1 contains mainly the receptor-binding domain (RBD), which is responsible for recognizing cell surface receptors. The S1 subunit specifically recognizes and binds to a cellular receptor (e.g. ACE2) and facilitates the fusion process together with the cell surface serine protease, another host factor (4). The successful replication of the virus and the success of the infection depend on the interaction of the virus-encoded protein with important host factors.

C-reactive protein (CRP) is a typical human acute-phase protein and a common clinical marker of inflammation and the plasma levels of CRP increase rapidly under inflammatory conditions (5, 6). CRP was originally identified by Tillet and Francis in their observations of patients with *Streptococcus pneumoniae* infection, where the serum of some acutely ill patients was bound to the cell wall capsule C-polysaccharide of *S. pneumoniae* to form a complex; this substance that was subsequently shown to precipitate with the C-polysaccharide was CRP (7). CRP, which is evolutionarily conserved, recognizes invading pathogens and apoptotic necrotic cells and further stimulates the classical pathway of the complement system to facilitate rapid clearance of these harmful substances (8, 9). Thus, CRP is often considered a putative pattern recognition receptor that plays an important role in inflammation and host defense (10). Further, pentameric CRP (pCRP) by binding to cell-derived microvesicles undergoes a structural change without disrupting the pentameric symmetry (pCRP*) (11). pCRP* constitutes the major CRP species in human-inflamed tissue and allows binding of complement factor 1q (C1q) and the activation of the classical complement pathway. pCRP*–microvesicle complexes lead to an enhanced recruitment of leukocytes to inflamed tissue.

Notably, elevation of CRP level is one of the main clinical features of patients with COVID-19, and high levels of CRP in the early stage of COVID-19 have been associated with lung damage and disease severity (12, 13). Analysis of lung alterations assessed using computed tomography shows that CRP level is high before the appearance of lung lesions, indicating that CRP levels may be used to predict COVID-19 disease severity (14). An elevated CRP/lymphocyte ratio has been used as an important predictor of the need for intensive care units (15, 16).

Recent studies have shown that pentameric CRP binding to pathogens or damaged cell membranes results in its conversion to an activated conformation (monomeric CRP, mCRP) that exhibits significantly enhanced ligand recognition and cellular stimulatory capacity (17–20). Our work and those of others have shown that the function of CRP depends strictly on its conformation and localization, the contribution of which varies with diseases (21, 22). Indeed, many of the proinflammatory activities of CRP are only observed in the inflammatory microenvironment where it is transformed to the activated state conformation (mCRP). The significantly enhanced ligand-binding capacity is key for the involvement of mCRP in the regulation of multiple inflammatory processes (23). In the present study, we investigated the mechanism underlying mCRP binding to the spike RBD and its cholesterol-binding sequence (CBS) motif and found that mCRP can bind to the SARS-CoV-2 spike RBD and competitively inhibit the binding of the spike RBD to human ACE2. Furthermore, the CBS motif (amino acids 35-47) in mCRP, which is the primary sequence responsible for interactions with the ligand, recognizes various molecules, including the spike RBD. The ability of the CBS motif to block the interactions between the spike RBD and ACE2 was investigated using flow cytometry in a constructed cell model of spike-ACE2 interactions. The findings are expected to provide new ideas for inflammatory intervention strategies targeting the CRP activation conformation.

## Materials and Methods

### Reagents

SARS-CoV spike RBD protein (catalog number: 40150-V05H) and recombinant human ACE2 protein (catalog number: 10108-H08H) were purchased from Sino Biological Inc (Beijing, China). Human native CRP purified from ascites was purchased from the Binding Site (Birmingham, UK; catalogue number: BP300.X). Lomefloxacin (catalog number: S5491) was procured from Selleck (USA). Urea-denatured mCRP (u-mCRP) and recombinant mCRP (r-mCRP) mutants were prepared as described (23, 24). Proteins were dialyzed to remove NaN3 and passed through Detoxi-Gel Columns (Thermo Fisher Scientific, Rockford, IL; catalog number: 20344) to remove endotoxin when necessary. CRP peptides were synthesized by Science Peptide Biologic Technology (Shanghai, China). Monomeric CRP mAbs 3H12 were generated as described (25).

### Spike RBD-mCRP Binding Assays

Surface plasmon resonance (SPR) binding assays were performed using Biacore 8K instrument (Cytiva Life Sciences, Marlborough, MA, USA) in HEPES buffer containing 50 mM HEPES (pH 7.4), 150 mM NaCl, and 0.05% Tween-20 at 25°C. Spike-RBD was diluted to a concentration of 20 μg/mL with 10 mM sodium acetate (pH 5.0) and immobilized on a CM5 sensor chip (Cytiva Life Sciences, Marlborough, MA, USA). The mCRP samples were passed over the sensor chip at a flow rate of 30 μL/min with a concentration gradient from 3.125 nM to 100 nM. The association time was 180 s. A dissociation process was performed for 200 s at a flow rate of 30 μL/min. The dissociation constant (Kd) values were calculated by fitting result curves (subtracted from the control flow value) to a 1:1 Langmuir binding model with Biacore 8K evaluation software.

The interactions of the spike-RBD with mCRP or CRP were also examined by enzyme-linked immunosorbent assay (ELISA) using mCRP/CRP-specific mAbs. Briefly, 100 μL of spike-RBD (1μg/mL) in Tris-buffered saline (TBS; 10 mM Tris, 140 mM NaCl, pH 7.4) was added per well to coat in 96-well microplates overnight at 4°C. The wells were then washed three times with TBS containing 0.02% Tween-20 for 5 min and blocked with 1% bovine serum albumin (BSA) in TBS for 1 h at 37°C. Next, mCRP was added and the plates were incubated for 1 h at 37°C. The color was developed by adding tetramethyl benzidine (TMB) to the wells and the color development was stopped with 1 M H_2_SO_4_.

### Peptide Competition Assay

For the peptide competition assay, microtiter wells were coated with the recombinant spike RBD (2μg/mL) overnight at 4°C. The wells were then washed three times with TBS (10 mM Tris, 140 mM NaCl, pH 7.4) containing 0.02% Nonidet P-40 and blocked with 1% BSA in TBS for 1 h. mCRP, at the half-saturation concentration of that used in the spike-mCRP binding assay with or without the indicated synthetic peptide, was then added and allowed to bind for 1 h. Competitive binding was detected using an mCRP-specific mAb (3H12, obtained from Dr. L.A.Potempa) and an HRP-conjugated Goat Anti-Mouse IgG (H+L) secondary antibody (Beyotime, Beijing, China; catalog number: A0216). After washing three times with TBS, the wells were developed using TMB and optical density was read at 450/570 nm.

### Expression and Purification of mCRP Mutants

Expression vectors of His-tagged wild type (WT) and mutant mCRP were induced in *E. coli* BL21 (DE3) by culture in a medium containing 0.5 mM isopropyl β- d-1-thiogalactopyranoside (IPTG) for 4 h at 37°C. Inclusion bodies were collected, washed, and solubilized overnight with 6 M guanidine hydrochloride (GuHCl) at 4°C. The supernatants were filtered twice with 0.22 μm membranes and purified by affinity chromatography using Ni-NTA resin (Qiagen, Hilden, Germany) and Superdex 200 prep grade column on an AKTA purification system (GE Life Sciences). The bound proteins were washed with phosphate-buffered saline (PBS) containing 40 mM imidazole and 6 M GuHCl, followed by elution with PBS containing 500 mM imidazole and 6 M GuHCl. The eluted proteins were regenerated, citraconylated, and kept in a buffer (pH 7.4) containing 10 mM Tris and 15 mM NaCl. The protein concentration was determined using the BCA method according to the manufacturer’s instructions.

### Inhibition Assay of Spike-ACE2 Interaction in Cells

First, HEK-293 cell line with a stable over-expression of 3× Flag-spike-RBD region was constructed by lentivirus infection. The spike-RBD protein was then purified from the cell lysates *via* Flag affinity chromatography. The purified spike-RBD protein (140 nM) was then pre-incubated with ACE2^+/+^ cells at 37°C for 1 h to construct the spike-ACE2 interaction cell model. The cells were then lifted and diluted in a flow cytometric staining buffer (PBS containing 1% FBS) to a final concentration of 2×10^7^ cells/mL. Different concentrations of CBS were added to the cell samples and the mixtures were incubated at 4°C for 30 min. After washing twice, the mixture was incubated with an anti-Flag antibody (1:500, catalog number: F7425; Sigma-Aldrich, St. Louis, MO, USA) at room temperature for another 30 min, followed by incubation with a secondary donkey anti-rabbit antibody conjugated to Alexa Fluor 488. Cellular fluorescence was quantitated as the mean fluorescence intensity or the percentage of positive cells.

The expression of hACE2 was determined using quantitative PCR with the primers (forward: 5′- TGTGGGATGGAGTACCGACT-3′; reverse: 5′-GCACATCCTCCTCCCCAAAA-3′). The sh-hACE2 sequences used for generating hACE2 knockdown cell lines were: sh-hACE2 (forward: 5′-CCGGGCTGGACAGAAACTGTTCAATCTCGAGATTGAACAGTTTCTGTCCAGCTTTTTG-3′; reverse: 5′-AATTCAAAAAGCTGGACAGAAACTGTTCAATCTCGAGATTGAACAGTTTCTGTCCAGC-3′). The inhibition capacity of CBS and positive control (lomefloxacin) was compared by flow cytometry using FITC-labeled spike-RBD and PE-labeled ACE2.

### Statistical Analysis

Data are presented as the mean ± SEM. Statistical analysis was performed by the two-tailed Student’s *t*-test or one-way ANOVA with Tukey’s *post hoc* test. Values of *p* < 0.05 were considered significant.

## Results

### mCRP Binds to the SARS-CoV-2 Spike RBD

**Figure 1:**
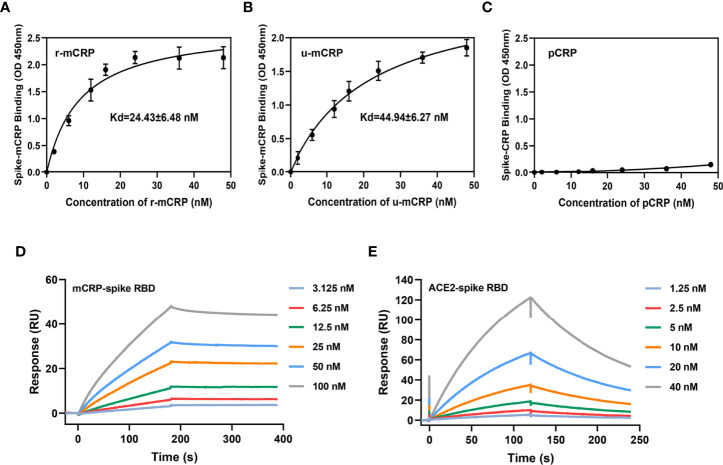
Binding of monomeric C-reactive protein (mCRP) to the spike receptor-binding domain (RBD). Different concentrations of **(A)** recombinant mCRP (r-mCRP) (n = 3), **(B)** urea-denatured mCRP (u-mCRP) (n = 3), and **(C)** pCRP were added to spike RBD-immobilized microtiter wells. Binding was detected with the mCRP-specific mAb 3H12 or the pCRP-specific mAb 8D8. mCRP obtained in different ways showed strong binding to the spike RBD, whereas pCRP did not (n = 3). **(D)** Surface plasmon resonance (SPR) assays to detect the binding of mCRP to the spike RBD. mCRP was injected in the fluid phase into spike RBD-conjugated to CM5 chips. CM5 chips without spike RBD conjugation were used as controls. Dose-dependent binding was observed with mCRP. **(E)** SPR assays to detect the binding of angiotensin-converting enzyme 2 (ACE2) to spike RBD. The binding of spike RBD to ACE2 was detected using SPR to determine the biological activity of spike RBD protein used in the binding experiment. All results are presented as means ± S.E.M.

We first used the purified RBD of the SARS-CoV-2 S protein to detect the specific binding between mCRP and RBD. The binding of mCRP or CRP to the spike RBD was detected using ELISA with an mCRP- or CRP- specific monoclonal antibody (mAb). The results showed that recombinant mCRP (r-mCRP) and urea-denatured CRP (u-mCRP) could specifically bind to the spike RBD (**Figures 1A, B**) and that pCRP did not bind to the spike RBD (**Figure 1C**). Furthermore, surface plasmon resonance (SPR) was used to verify the binding of mCRP and the spike RBD. The results of SPR also showed that mCRP could bind to the spike RBD with high affinity (**Figure 1D**). Finally, SPR was used to verify the biological binding of the spike RBD to ACE2 and to determine the activity of the spike RBD protein that was used in the abovementioned experiments. The results showed that the spike RBD could bind to its receptor ACE2 with high affinity (**Figure 1E**), indicating that the spike RBD protein used in the abovementioned binding experiments possessed biological activity.

### mCRP Competes for the Binding of Spike RBD to ACE2

**Figure 2:**
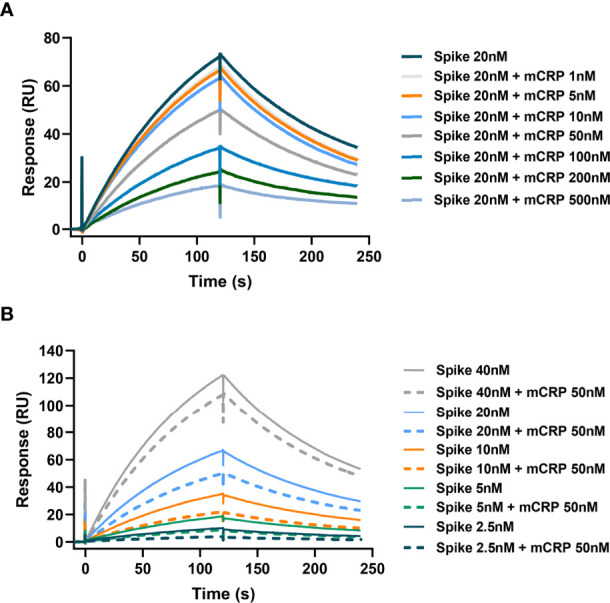
mCRP competitively inhibits the binding of the spike RBD to ACE2. **(A)** mCRP competes for the binding of spike RBD to ACE2 in a concentration-dependent manner. In the surface plasmon resonance (SPR) binding assay, different concentrations of mCRP were added to compete for the binding of the spike RBD to ACE2. Results showed that mCRP competed for the binding of the spike RBD to ACE2 in a concentration-dependent manner. **(B)** A fixed concentration of mCRP competes with the binding of different concentrations of the spike RBD to ACE2. For detecting the binding of different concentrations of the spike RBD to ACE2 (solid line), a fixed concentration of mCRP was added to compete for binding (dotted line), and mCRP competitively inhibited the binding of the spike RBD to ACE2.

Having confirmed that mCRP can bind to the spike RBD, we further questioned whether mCRP can compete for the binding of the spike RBD to ACE2. For this, we performed SPR to determine whether mCRP competitively inhibited the binding of spike RBD to ACE2. First, in the spike RBD binding assay with ACE2, the spike RBD concentration was fixed and different concentrations of mCRP were added to test whether mCRP could inhibit the binding of the spike RBD to ACE2. The results showed that mCRP significantly inhibited the binding of the spike RBD to ACE2 in a concentration-dependent manner (**Figure 2A**). We then used different concentrations of the spike RBD protein to compete with a fixed concentration of mCRP for binding; results showed that the addition of a certain concentration of mCRP inhibited the binding of the spike RBD to ACE2 to a certain extent at all the concentrations tested (**Figure 2B**). These results demonstrate that mCRP can competitively inhibit the binding of the spike RBD to ACE2.

### mCRP-Derived Peptide Competition Assay

**Figure 3:**
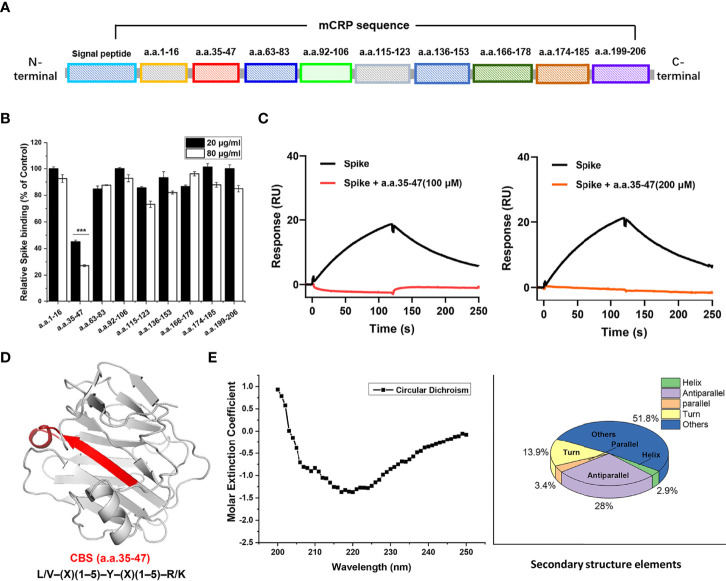
The 35-47 amino acid motif of cholesterol-binding sequence (CBS) mediates the binding of mCRP to the spike RBD. **(A)** Schematic showing the screening for the motif that mediates the binding of mCRP to the spike RBD. Full-length mCRP was truncated and peptides were synthesized. **(B)** Competitive inhibition assay to assess mCRP binding to the spike RBD using mCRP-derived synthetic peptides. In the peptide competition assay, amino acids 35-47 significantly inhibited the binding of mCRP to the spike RBD, whereas other sequences did not (n = 3). **(C)** Competitive inhibition assay to assess the binding of different concentrations of amino acids 35-47 mCRP to the spike RBD detected using SPR. The results showed that amino acids 35-47 significantly inhibited the binding of mCRP to the spike RBD. **(D)** Schematic representation of amino acids 35-47 in the crystal structure of mCRP. This sequence is consistent with the features of the CBS consensus harboring the L/V–(X)(1–5)–Y–(X)(1–5)–R/K motif. **(E)** The secondary structure of amino acids 35-47 in solution was detected using circular dichroism. All results are presented as means ± S.E.M.

mCRP contains some typical secondary structures and lacks tertiary structures (in spectral analysis, data not shown). Hence, we inferred that synthetic-truncated peptides derived from mCRP linear sequence motifs may retain their biological activity. We synthesized a series of peptides derived from the mCRP sequence (**Figure 3A**), which basically covered the full length of the mCRP protein, and examined whether they could competitively inhibit the binding of mCRP to the spike RBD. Peptide competition experiments showed that amino acids 35-47 (CBS) competitively inhibited the binding of mCRP to the spike RBD, whereas no inhibition was observed for other peptides (**Figure 3B**). SPR showed that different concentrations of the 35-47 amino acid peptide almost completely inhibited the binding of the spike RBD to mCRP (**Figure 3C**). We further analyzed the secondary structure of the 35-47 amino acid peptide in solution using circular dichroism spectroscopy and found that its secondary structure mainly consisted of β-sheets and unstructured elements (turns and coils) (**Figures 3D, E**).

### N-terminal Residues Determined the Performance of the CBS Peptide

**Figure 4:**
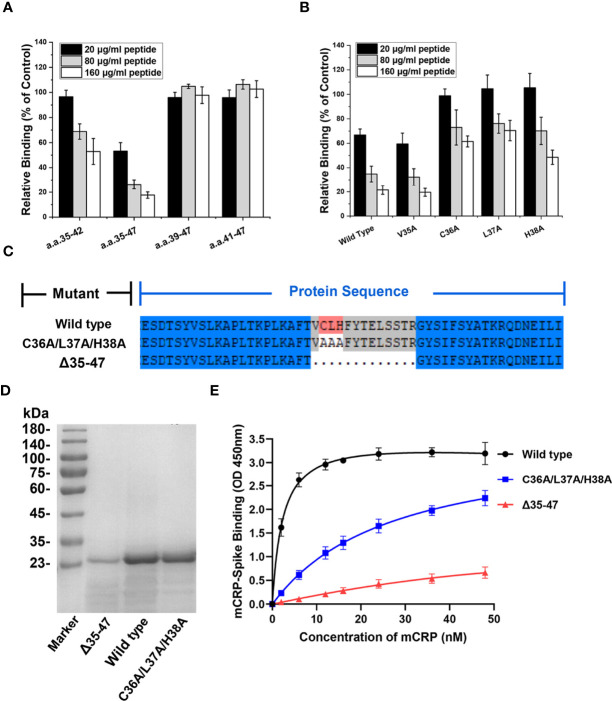
The N-terminal residues determine the ligand-binding ability of the CBS. **(A)** CBS mutants with truncations in its N or C terminus were tested for their effects on mCRP binding to the spike RBD (n = 3). The inhibitory effects of CBS were lost following N-terminal truncation but not C-terminal truncation. **(B)** CBS with point mutations at the indicated N-terminal residues were tested for their effects on mCRP binding to the spike RBD. The three amino acid point mutations at the N-terminus (C36A/L37A/H38A) significantly weakened the ability of the CBS to compete for mCRP and spike RBD binding (n = 3). **(C)** Schematic showing construction of mCRP 35-47 amino acid deletion mutants (Δ35-47) and C36A/L37A/H38A mutants. **(D)** The wild type, C36A/L37A/H38A mutant, and Δ35–47 mutants were constructed in *E. coli* and purified using SEC and SDS-PAGE. **(E)** mCRP wild type or mutants with altered CBS were expressed in *E. coli* and purified to test their binding to the spike RBD (n = 3). The binding of mCRP mutants was impaired by mutating the 3 N-terminal residues (C36A/L37A/H38A) or deleting amino acids 35–47. All results are presented as means ± S.E.M.

After identifying CBS (amino acids 35−47) as the key sequence mediating mCRP-spike RBD binding, we synthesized a series of N- or C-terminus truncated mutants of the CBS and evaluated their inhibitory effect. Truncated mutants without the N-terminus but with the C-terminus, such as amino acids 39−47 and 41−47, lost the ability to inhibit mCRP binding to the spike RBD. However, truncated mutants that retained the N-terminus but lacked the C-terminus, such as amino acids 35−42, still inhibited the spike RBD binding to mCRP (**Figure 4A**). Therefore, the N-terminal sequence is critical for maintaining the CBS activity.

Next, alanine scanning was performed to delineate the contributions of the N-terminal residues. Four N-terminal amino acids, Val35, Cys36, Leu37, and His38, were individually mutated to Ala, and the inhibitory activity of CBS peptides harboring the N-terminus amino acid point mutations was detected. The results showed that the point mutations of Cys36, Leu37, and His38 considerably affected the ability of the CBS to inhibit the binding of the spike RBD to mCRP, whereas the point mutation of Val35 showed a weak effect (**Figure 4B**). This suggested that the N-terminal Cys36, Leu37, and His38 are critical for maintaining the inhibitory activity of the CBS. In addition, Cys36 of mCRP is also key for the formation of disulfide bonds, suggesting that subunit disulfide bonds regulate the effectiveness of mCRP-mediated inhibition of the RBD binding to ACE2.

After confirming that amino acids 35−47 are important for mediating the interaction between the spike RBD and mCRP, we constructed and purified mutants with deletion of amino acids 35−47 (Δ 35-47) or their point mutations Cys36Ala/Leu37Ala/His38Ala (**Figures 4C, D**). Results of ELISA with WT and mutant proteins showed that WT mCRP was bound to the spike RBD, whereas binding of mCRP to the spike RBD was impaired in the Cys36Ala/Leu37Ala/His38Ala mutant; deletion of residues 35−47 almost abrogated mCRP binding (**Figure 4E**). Together, the abovementioned results demonstrated that the specificity and potency of amino acids 35−47 are determined by Cys36, Leu37, and His38 in the N-terminal of CBS.

### Inhibition of Spike RBD-ACE2 Interaction in Cells

**Figure 5:**
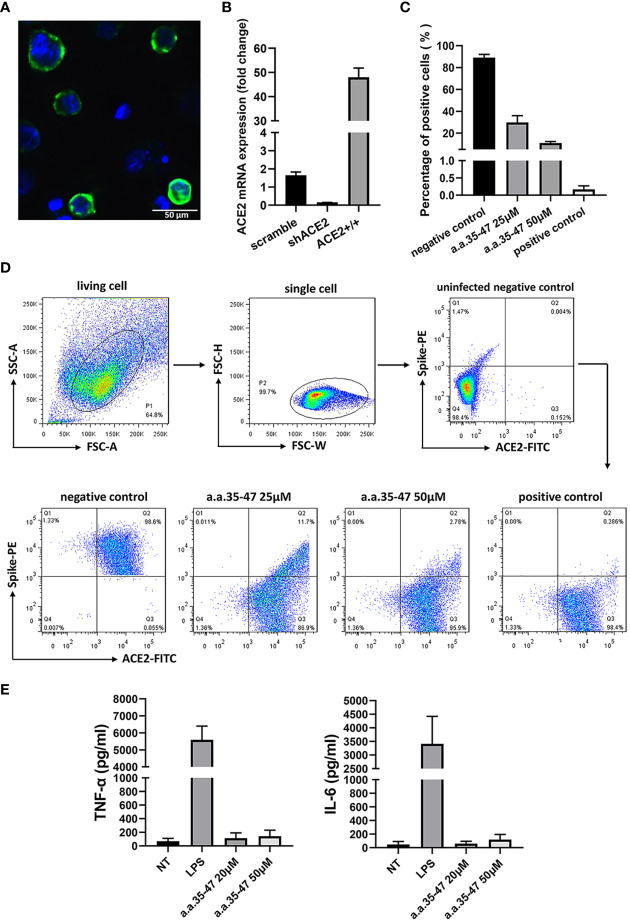
CBS inhibits the spike RBD from binding to cell surface ACE2. **(A)** Immunofluorescence of the A549 cells stably expressing ACE2. Green fluorescence indicates ACE2-mNEOGreen and blue indicates DAPI. **(B)** mRNA expression of ACE2-overexpressing (ACE2+/+) and knockdown (shACE2) cell lines (n = 3). **(C)** Results of flow cytometry showing that CBS inhibits the interaction between the spike RBD and ACE2 at the cellular level (n = 3). Lomefloxacin acted as the positive control and untreated cells served as the negative control. **(D)** Schematic showing the flow cytometry gating process and typical flow cytometry diagrams of different concentrations of CBS-treated cells and controls. **(E)** CBS itself did not induce the release of cytokines from immune cells. Immortalized bone marrow-derived macrophages (iBMDM) were stimulated with different concentrations of CBS, and lipopolysaccharide (LPS) was used as the positive control (n = 3). Results showed that the CBS itself did not stimulate cells. All results are presented as means ± S.E.M.

In the cell assay for determining spike-ACE2 interaction, a HEK 293T cell line stably expressing the 3× Flag-tagged-spike RBD region was constructed *via* lentivirus infection, and the spike RBD was purified from the cell lysate using Flag affinity chromatography. Next, we constructed an A549 cell line stably overexpressing tdTomato-tagged-hACE2, and the purified spike RBD protein (140 nM) was co-incubated with ACE2^+/+^ cell line to construct the spike-ACE2 interaction cell model (**Figures 5A, B**).

To compare the inhibitory ability of the CBS peptide, different concentrations of the CBS peptide were added to the cell medium and incubated at 4°C for 30 min. After washing, the mixture was incubated for another 30 min with anti-Flag antibody, followed by incubation with a secondary antibody conjugated to Alexa Fluor 488. The inhibitory activity of the CBS peptide and positive control (lomefloxacin) was compared using flow cytometry with Alexa Fluor 488-conjugated spike RBD and phycoerythrin (PE)-conjugated ACE2. Results showed that the CBS peptide could inhibit the binding of the spike RBD to the cell surface ACE2 in a concentration-dependent manner; the positive control, lomefloxacin, almost completely inhibited the binding, thereby confirming the spike-ACE2 interaction in the interaction cell model (**Figures 5C, D**). At the same time, we stimulated macrophages with the CBS peptide and detected the release of pro-inflammatory cytokines TNF-α and IL-6 to verify whether the CBS peptide itself is immunogenic. Results showed that the CBS peptide itself did not induce the release of cytokines (**Figure 5E**).

### Conservation Analysis and Molecular Dynamics (MD) Simulation of CBS

**Figure 6:**
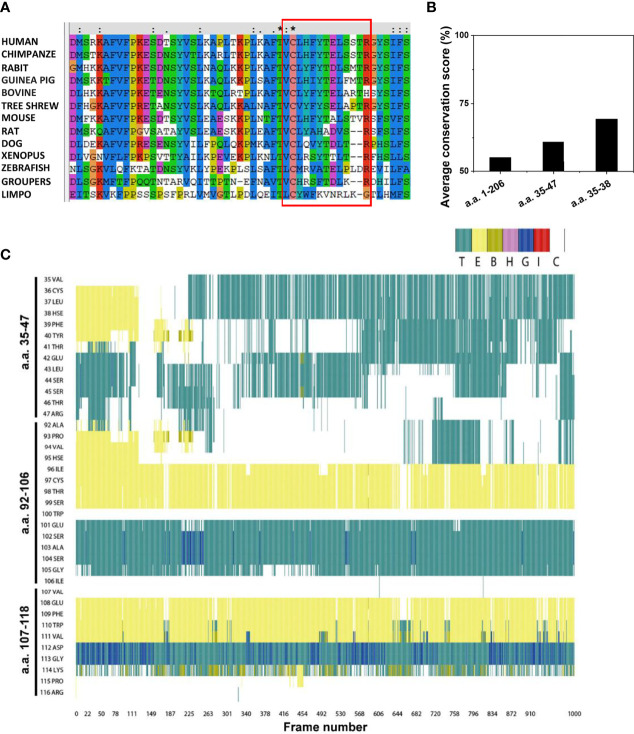
Conservation analysis and molecular dynamics simulation of the CBS. **(A)** Amino acid sequences of CRP from various species were retrieved from the UniProt database and analyzed using the Clustal X web services and DNAMAN software. The CBS motifs in different species are shown using a red box. **(B)** Average conservation scores of full-length CRP (amino acids 1-206), CBS (amino acids 35-47), and the N-terminal of CBS (amino acids 35-38). **(C)** Frequent oscillation to and from coiled conformation of CBS. The conformations of all residues in the CBS (amino acids 35-47), amino acids 92-106, and amino acids 107-118 at each frame (0.1 ns) in the same 100-ns simulation are shown. E, B, and T represent different styles of β-sheet conformation; H, G, and I represent different types of helix conformation; C represents coiled conformation.

We then selected mCRP sequences from different species, performed sequence alignment, and detected whether the CBS sequence pattern was evolutionarily conserved (**Figure 6A**). Consistent with its functional importance, the full-length CBS, particularly its N-terminal portion, exhibited strong evolutionary conservation (**Figure 6B**). Among the N-terminal residues, Cys36 was highly conserved and was involved in ligand binding. As Cys36 normally forms an intra-subunit disulfide bond with Cys97, this highlights the importance of the redox status of Cys36 in tuning the activities of the CBS.

We performed MD simulations on the CBS and control peptides to determine the possible structural features of the CBS (**Figure 6C**). The initial structures of CRP peptides were obtained from the crystal structure (PDB 1B09). The CBS and control peptides were immersed together in the explicit solvate box of 57 × 50 × 52 Å^3^ dimension using a TIP3P water model with infinite periodic boundary conditions. Results showed that the CBS sequence had more coiled domains, which is characteristic of intrinsic disorder. In contrast, the conformations of two control CRP peptides, spanning amino acids 92–106 (with structural features similar to CBS) and 107–118, were stable during the same simulation. Some intrinsic disordered motifs do not have any defined three-dimensional structure but tend to have localized regions of stable secondary structures that are often binding sites for protein-ligand interactions and may be the initiation sites for the binding process.

## Discussion

CRP, which belongs to the pentraxin family, is assembled from five identical subunits *via* non-covalent bonds (26). CRP is ubiquitous in vertebrates and invertebrates. Its structure and sequence are evolutionarily conserved, suggesting that this ancient protein performs important biological functions (27). Increase in CRP plasma level is one of the main clinical characteristics of patients with COVID-19 (12–14), which indicates that in addition to being a marker of inflammation, CRP may be involved in the diagnosis and pathogenesis of COVID-19. Our previous experimental results showed that CRP exerted a protective effect on acute lung injury. Mice with CRP knockout were more sensitive to acute lung injury than WT mice, showing more severe cytokine release and tissue injury (data not shown). However, whether CRP is involved in the regulation of COVID-19 pneumonia and its potential mechanism of action remain unknown.

Recent studies conducted by our group and others have revealed that the pro-inflammatory activity of CRP is mainly because of the mCRP produced *via* dissociation in local inflammatory lesions, rather than because of its pentamer form, indicating that the function of CRP depends on its conformation and spatial localization (21, 22). The role of mCRP in infection is not completely understood; specifically, the reason behind the improvement in the recognition and binding ability of mCRP with ligands after the dissociation of CRP is unclear. Because of the challenges in crystallizing mCRP, its high-resolution crystal structure has not been reported, which hinders the identification of the corresponding ligand recognition sites as well as the designing of specific targets and interventions. However, mCRP contains a few secondary structures and lacks a tertiary structure, suggesting that potential functional peptides containing recognition motifs can be screened based on its linear amino acid sequence.

In this study, we found that mCRP can bind to the SARS-CoV-2 spike RBD and compete with the binding of the spike RBD to ACE2 in a concentration-dependent manner. Using this as a starting point and combined with the structural characteristics of mCRP, truncated mutant peptides were systematically prepared, and the key motifs responsible for the recognition of mCRP to the spike RBD were screened in the full-length linear sequence. Results of the peptide competition assay showed that the synthetic peptides encompassing amino acids 35−47 of mCRP competitively inhibited the interaction between mCRP and the spike RBD. To exclude the possibility of artifacts caused by experimental errors, SPR competition experiments were further used for verification and the same results were obtained. In addition, the ability to bind to ligands was significantly weakened in the 35−47 deletion mutant protein (△35−47). Experiments with point mutants showed that the N-terminus of the 35−47 motif was critical for this function, strongly suggesting that amino acids 35−47 may be responsible for the recognition of the spike RBD in mCRP and that the corresponding sequence of the synthetic peptide may be a conformation-specific functional motif.

The amino acid sequence from positions 35 to 47 was further aligned and found to be consistent with features of the cholesterol recognition/interaction consensus motif, L/V–(X)(1–5)–Y–(X)(1–5)–R/K (28). Results of the multi-species alignment analysis revealed that CBS in CRP was conserved in most species, which was only exposed when the pCRP dissociated to mCRP. Intrinsically disorder is another structural feature of the CBS in mCRP. Although disordered motifs do not have any defined three-dimensional structure, they tend to possess localized regions of stable secondary structures that are often binding sites for protein-ligand interactions and may act as initiation sites for the binding process (29). Known as the molecular recognition motif, the CBS sequence spanning 35-47 amino acids in mCRP is possibly an inherently disordered recognition site responsible for mutual recognition of its ligands.

SARS-CoV-2 infection induces high levels of CRP, potentially causing uncontrolled autoimmune responses. There are case reports showing that the clearance of CRP in serum can reduce the immune response (30, 31). CRP-mediated activation of complement and macrophages is suspected to be a driver of human pulmonary fibrosis and subsequent organ failure (31). Regarding the multifaceted role of CRP, the effects of CRP may differ at different infection stages. CRP is moderately elevated in the early stage of infection and plays the role of a soluble pattern recognition receptor, which is conducive to the recognition of necrotic cells or damaged tissues; however, in the middle and late stages of infection, uncontrollably high levels of CRP reflect the severity of the infection and may cause autoimmunity that promotes inflammation and complications. Furthermore, mCRP is generated primarily within inflamed tissues (19, 21) and the half-life of blood-borne mCRP is rather short (21), it appears conceivable that circulating mCRP does not play a significant role in the direct induction of autoimmune responses.

Our study has certain limitations. First, the inhibition of binding is observed at relatively high concentrations of the CBS motif, and peptide modification is required to increase the effective unit concentration and to improve its delivery. Second, under physiological conditions, SARS-CoV-2 is a highly glycosylated virus (32), and its ACE2-binding site may be shielded by glycosylation (33, 34), which has to be considered in subsequent applications. Third, the current work mainly focuses on the physical binding and competitive inhibition in *in vitro* systems, while functional experiments are still lacking. We have used a SARS-CoV-2 pseudovirus system (35) to test the ability of the CBS in mCRP (a.a.35-47) to inhibit the pseudovirus infection of cells for functional verification. Our preliminary results showed that a.a.35-47 (50 μM) could partially inhibit the invasion of SARS-CoV-2 pseudovirus into Caco-2 cells (data not shown). Nevertheless, further improvements of the functional tests and *in vivo* experiments are an important focus area of our follow-up work.

## Data Availability Statement

The raw data supporting the conclusions of this article will be made available by the authors, without undue reservation.

## Author Contributions

H-yL and Q-yL designed the research. H-yL, NG, C-yL, X-lL, ND, and HJ performed the research. H-yL, Q-yL, C-yL, and FW analyzed the data and wrote the paper. All authors reviewed the results and approved the final version of the manuscript.

## Funding

This work was supported by the Science Foundation for COVID-19 of Xi’an Jiaotong University and Qinnong Bank (QNXJTU-08); the National Natural Science Foundation of China (81900641); the Research Funding of Peking University (BMU2021MX020, BMU2022MX008) and the Natural Science Foundation of Shaanxi Province [grant number 2020JQ-025].

## Conflict of Interest

The authors declare that the research was conducted in the absence of any commercial or financial relationships that could be construed as a potential conflict of interest.

## Publisher’s Note

All claims expressed in this article are solely those of the authors and do not necessarily represent those of their affiliated organizations, or those of the publisher, the editors and the reviewers. Any product that may be evaluated in this article, or claim that may be made by its manufacturer, is not guaranteed or endorsed by the publisher.

## Abbreviations

ACE2, angiotensin-converting enzyme 2; CBS, cholesterol-binding sequence; COVID-19, coronavirus disease 19; SARS-CoV-2, severe acute respiratory disease coronavirus 2; mCRP, monomeric C-reactive protein; RBD, receptor-binding domain.
