# Corrigendum: The cholesterol-binding sequence in monomeric C-reactive protein binds to the SARS-CoV-2 spike receptor-binding domain and blocks interaction with Angiotensin-converting enzyme 2

**DOI:** 10.3389/fimmu.2022.1011789

**Published:** 2022-09-12

**Authors:** Hai-yun Li, Ning Gao, Cheng-yang Liu, Xiao-ling Liu, Feng Wu, Nini Dai, Jing Han, Qiu-yu Li

**Affiliations:** ^1^ Ministry of Education (MOE) Key Laboratory of Environment and Genes Related to Diseases, School of Basic Medical Sciences, Xi’an Jiaotong University, Xi’an, China; ^2^ Department of Biochemistry and Molecular Biology, School of Basic Medical Sciences, Xi’an Jiaotong University, Xi’an, China; ^3^ Department of Infectious Disease, the Second Affiliated Hospital of Xi’an Jiaotong University, Xi’an, China; ^4^ Academy for Advanced Interdisciplinary Studies, Peking University, Beijing, China; ^5^ Ministry of Education (MOE) Key Laboratory of Cell Activities and Stress Adaptations, School of Life Sciences, Lanzhou University, Lanzhou, China; ^6^ Center of Teaching and Experiment for Medical Post Graduates, School of Medicine, Xian Jiotong University, Xian, China; ^7^ Department of Respiratory and Critical Care Medicine, Peking University Third Hospital, Beijing, China

**Keywords:** SARS-CoV-2, monomeric C-reactive protein, pattern recognition receptor, ACE2, cholesterol-binding sequence

In the original article, there was a mistake in **Figure 5D** as published. The positive control of **Figure 5D** was misplaced. The positive control of **Figure 5D** has been replaced with the correct version. The corrected **Figure 5D** and its caption appear below.

**Figure 5:**
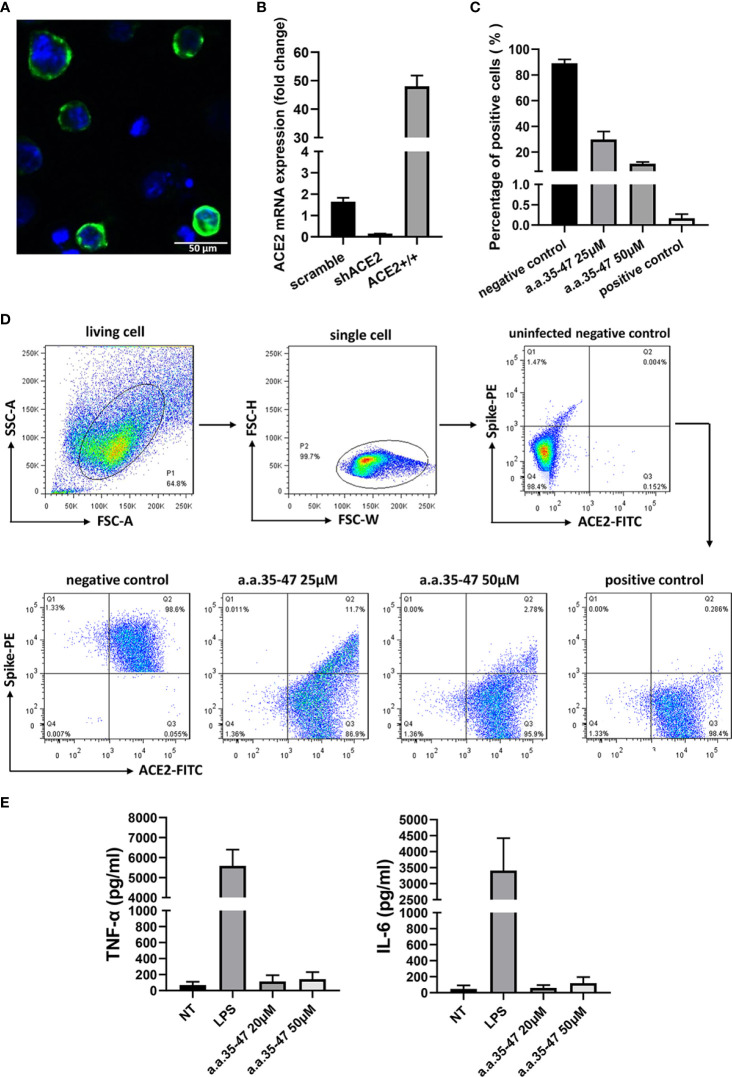
CBS inhibits the spike RBD from binding to cell surface ACE2. **(A)** Immunofluorescence of the A549 cells stably expressing ACE2. Green fluorescence indicates ACE2-mNEOGreen and blue indicates DAPI. **(B)** mRNA expression of ACE2-overexpressing (ACE2+/+) and knockdown (shACE2) cell lines (n = 3). **(C)** Results of flow cytometry showing that CBS inhibits the interaction between the spike RBD and ACE2 at the cellular level (n = 3). Lomefloxacin acted as the positive control and untreated cells served as the negative control. **(D)** Schematic showing the flow cytometry gating process and typical flow cytometry diagrams of different concentrations of CBS-treated cells and controls. **(E)** CBS itself did not induce the release of cytokines from immune cells. Immortalized bone marrow-derived macrophages (iBMDM) were stimulated with different concentrations of CBS, and lipopolysaccharide (LPS) was used as the positive control (n = 3). Results showed that the CBS itself did not stimulate cells. All results are presented as means ± S.E.M.

The authors apologize for this error and state that this does not change the scientific conclusions of the article in any way.

## Publisher’s note

All claims expressed in this article are solely those of the authors and do not necessarily represent those of their affiliated organizations, or those of the publisher, the editors and the reviewers. Any product that may be evaluated in this article, or claim that may be made by its manufacturer, is not guaranteed or endorsed by the publisher.

